# Unveiling the Forensic Potential of Oral and Nasal Microbiota in Post-Mortem Interval Estimation

**DOI:** 10.3390/ijms26073432

**Published:** 2025-04-06

**Authors:** Ji Chen, Qi Wei, Fan Yang, Yanan Liu, Yurong Zhao, Han Zhang, Xin Huang, Jianye Zeng, Xiang Wang, Suhua Zhang

**Affiliations:** 1 Institute of Forensic Science, Fudan University, Shanghai 200032, China; chenji@fudan.edu.cn (J.C.); wqwfmxlh@163.com (Q.W.); 23111010069@m.fudan.edu.cn (X.H.); 23111010090@m.fudan.edu.cn (J.Z.); 2 Ministry of Education’s Key Laboratory of Contemporary Anthropology, School of Life Sciences, Fudan University, Shanghai 200438, China; fan_yang_27@163.com (F.Y.); dyndai@sina.cn (Y.L.); 3 Key Laboratory of Forensic Evidence and Science Technology, Institute of Forensic Science, Ministry of Public Security, Shanghai 200042, China; 4 School of Life Sciences, Fudan University, Shanghai 200438, China; zyrguanghuanfa@outlook.com; 5 Department of Forensic Medicine, Guizhou Medical University, Guiyang 550004, China; zhang_h@stu.gmc.edu.cn

**Keywords:** 16S rRNA, microbial communities, post-mortem interval, random forest model, freezing

## Abstract

Microbiota have emerged as a promising tool for estimating the post-mortem interval (PMI) in forensic investigations. The role of oral and nasal microbiota in cadaver decomposition is crucial; however, their distribution across human cadavers at different PMIs remains underexplored. In this study, we collected 88 swab samples from the oral and nasal cavities of 10 healthy volunteers and 34 human cadavers. Using 16S rRNA gene sequencing, we conducted comprehensive analyses of the alpha diversity, beta diversity, and relative abundance distribution to characterize the microbial communities in both healthy individuals and cadavers at varying PMIs and under different freezing conditions. Random forest models identified *Firmicutes*, *Proteobacteria*, *Bacteroidota*, *Actinobacteriota*, and *Fusobacteriota* as potential PMI-associated biomarkers at the phylum level for both the oral and nasal groups, along with genus-level biomarkers specific to each group. These biomarkers exhibited nonlinear changes over increasing PMI, with turning points observed on days 5, 12, and 22. The random forest inference models demonstrated that oral biomarkers at both the genus and phylum levels achieved the lowest mean absolute error (MAE) values in the training dataset (MAE = 2.16 days) and the testing dataset (MAE = 5.14 days). Additionally, freezing had minimal impact on the overall phylum-level microbial composition, although it did affect the relative abundance of certain phyla. At the genus level, significant differences in microbial biomarkers were observed between frozen and unfrozen cadavers, with the oral group showing greater stability compared to the nasal group. These findings suggest that the influence of freezing should be considered when using genus-level microbial data to estimate PMIs. Overall, our results highlight the potential of oral and nasal microbiota as robust tools for PMI estimation and emphasize the need for further research to refine predictive models and explore the environmental factors shaping microbial dynamics.

## 1. Introduction

The post-mortem interval (PMI), defined as the time elapsed between death and the examination of the cadaver, is a crucial parameter in forensic investigations [1]. Traditional methods for PMI estimation, such as assessing rigor mortis, algor mortis, livor mortis, cadaveric discoloration, and gastric content digestion, are often affected by external environmental factors and rely heavily on the subjective judgment of forensic examiners [2,3,4]. To enhance PMI estimation, advanced techniques, including forensic entomology [5], molecular biology (e.g., analyses of RNA, DNA, and proteins) [6,7,8], spectroscopy [9], and radiological technologies [10], have been developed. However, these methods often suffer from drawbacks, such as limited precision, sensitivity to external environmental conditions, or inefficacy for longer PMIs [3]. Consequently, the development of more precise, reliable, and stable methods for PMI estimation remains a key focus in forensic research.

Recent studies have shown that microbial communities, both within and outside the body, undergo predictable and systematic changes after death, which are closely linked to the PMI [11,12,13,14,15]. Notably, integrating microbiome data with machine learning models has significantly improved the accuracy of PMI prediction [4,16,17,18]. These findings highlight that microbiome-based methodologies could offer a less subjective and potentially more reliable framework for PMI estimation.

Diverse microbiota residing in the oral and nasal cavities play important roles in cadaver decomposition, making them promising targets for PMI estimation research [19]. The ease and non-invasive nature of sample collection from these sites further strengthens their potential for forensic applications. Several studies have explored the relationship between microbial communities in the oral or nasal cavities and PMIs [17,20,21,22,23]. However, there are some limitations in the existing research. One notable issue is the relatively small number of studies involving large-scale human samples. Much of the current research relies on animal models, such as rats [17,20] and mice [4], which may not fully capture the complexity of human decomposition due to differences in physiology and microbial communities. Additionally, many human studies are limited by small sample sizes [21] or narrow PMI ranges [21], which can undermine the generalizability and accuracy of the findings. Moreover, research on the role of the nasal microbiota in PMI estimation remains sparse, and there has been relatively little investigation into how environmental factors, such as freezing, influence PMI estimation, highlighting key areas for further exploration [24,25,26].

This study aimed to examine the distribution of oral and nasal microbiota in both healthy living individuals and human cadavers, considering varying PMIs and different freezing conditions. We expected to uncover identifiable patterns and biomarkers within these microbiota that could be instrumental in estimating PMIs, providing valuable insights for forensic applications.

## 2. Results

### 2.1. Overall Characterization of 16S rRNA Sequencing Data

A total of 88 swab samples were collected and analyzed from the oral and nasal cavities of 10 healthy volunteers and 34 human cadavers. The 10 healthy volunteers (Group H) comprised 5 males and 5 females, with a mean age of 26.3 years. Among the 34 cadavers, there were 26 males and 8 females, with a mean age of 52.6 years. The PMI in this study was defined as the time elapsed from death to sample collection. Based on the stages of cadaveric decomposition [14], the cadavers were categorized into four groups: Group D1 (PMI 1–3 days, fresh decay stage), Group D2 (PMI 4–7 days, bloat stage), Group D3 (PMI 8–15 days, active decay stage), and Group D4 (PMI > 15 days, advanced decay stage). Detailed information is provided in Table 1.

All samples were successfully sequenced on an Illumina platform targeting the V3–V4 region of the 16S rRNA gene, generating a total of 7,277,506 raw reads. After removing low-quality reads, we obtained 6,376,368 clean reads and identified 8253 bacterial ASV sequences for the subsequent analysis. The average number of clean reads was 75,586 and the average number of ASVs was 293 per sample.

### 2.2. Microbial Diversity

**Figure 1:**
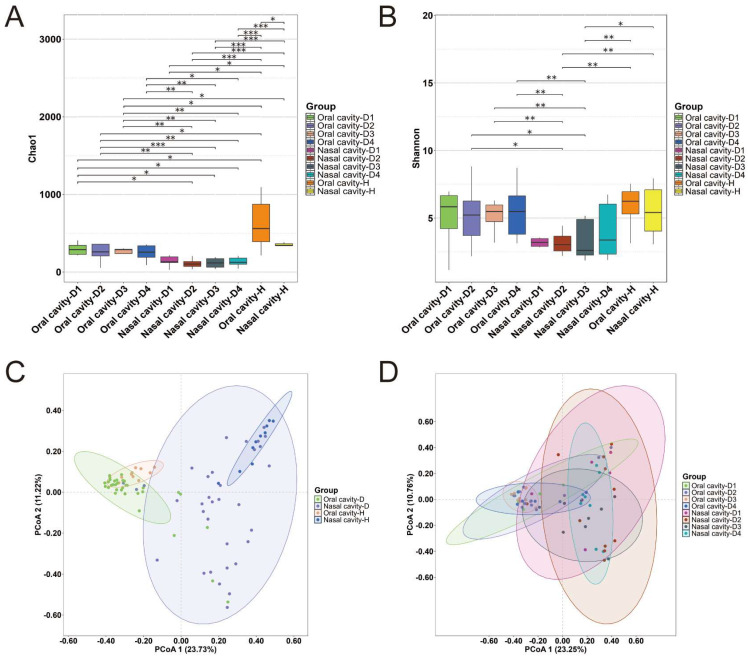
Analysis of microbial diversity among the studied sample groups. (**A**) Comparison of Chao1 index (alpha diversity) across groups. (**B**) Comparison of Shannon index (alpha diversity) across groups. (**C**,**D**) Beta diversity PCoA plots of the studied groups (*** *p* < 0.001, ** *p* < 0.01, * *p* < 0.05).

To evaluate the microbial diversity within groups (alpha diversity), we assessed the richness and evenness of the microbial communities using the Chao1 and Shannon indices at the amplicon sequence variant (ASV) level. As shown in Figure 1A, the Chao1 index indicated significantly higher species richness in the healthy volunteer groups than in the cadaver groups (*p* < 0.05). Regardless of the group type, the oral samples exhibited greater richness than the nasal samples. The Shannon index (Figure 1B) was lower in the nasal samples than in the oral samples. Furthermore, both indices fluctuated with increasing PMI but showed no consistent trend.

To assess the inter-group microbial diversity (beta diversity), a principal coordinates analysis (PCoA) based on Bray–Curtis distances was performed and visualized in two-dimensional space (Figure 1C,D). The clustering patterns revealed significant differences between the oral and nasal samples, especially among the healthy volunteer groups (Figure 1C). In contrast, no significant clustering was observed across the different PMI groups within either the oral or nasal sample sets (Figure 1D).

### 2.3. Microbial Abundance

The relative abundance of microbial communities across the 10 groups was analyzed at both the phylum and genus levels.

**Figure 2:**
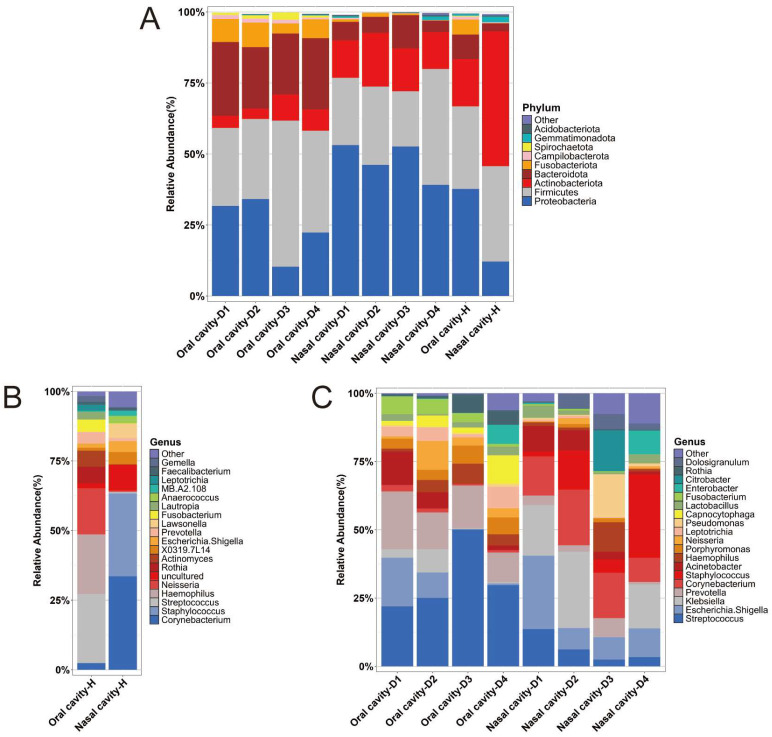
Composition and relative abundances of dominant microbial communities at the phylum and genus levels. (**A**) Relative abundance of the dominant microbial phyla across all the groups. (**B**) Relative abundance at the genus level within the healthy volunteer groups. (**C**) Relative abundance at the genus level within the cadaver groups.

At the phylum level, *Proteobacteria*, *Firmicutes*, and *Bacteroidota* were predominant in the oral cadaver groups (Oral cavity-D1, Oral cavity-D2, Oral cavity-D3, and Oral cavity-D4). In the nasal cadaver groups (Nasal cavity-D1, Nasal cavity-D2, Nasal cavity-D3, and Nasal cavity-D4), *Proteobacteria*, *Firmicutes*, and *Actinobacteriota* dominated, with *Proteobacteria* showing a higher relative abundance than *Firmicutes*. In the healthy volunteer groups (Oral cavity-H and Nasal cavity-H), the relative abundance of *Actinobacteriota* was higher compared to that of the corresponding oral or nasal cadaver groups. Additionally, the Nasal cavity-H group exhibited a lower relative abundance of *Proteobacteria* than the nasal cadaver groups (Figure 2A).

At the genus level, *Streptococcus* and *Haemophilus* were the predominant genera in the Oral cavity-H group, while *Corynebacterium* and *Staphylococcus* were notably more abundant in the Nasal cavity-H group (Figure 2B). In the oral cadaver groups, *Streptococcus* and *Prevotella* were the predominant genera. The nasal cadaver groups displayed variation in the dominant genera across different PMI stages: *Escherichia-Shigella* was the most abundant genus in Group D1, *Klebsiella* in Group D2, *Corynebacterium* in Group D3, and *Staphylococcus* in Group D4 (Figure 2C).

### 2.4. Biomarkers Associated with PMIs

To identify potential biomarkers for different PMIs in the oral or nasal cadaver groups, random forest models were constructed using the samples’ PMIs and the relative abundances of bacterial taxa at both the phylum and genus levels. The bacterial taxa were ranked based on their mean decrease in Gini values. Among the top 10 taxa, five with higher relative abundances were identified as promising biomarkers for PMIs. Subsequently, LOESS regression curves were produced for each biomarker.

**Figure 3:**
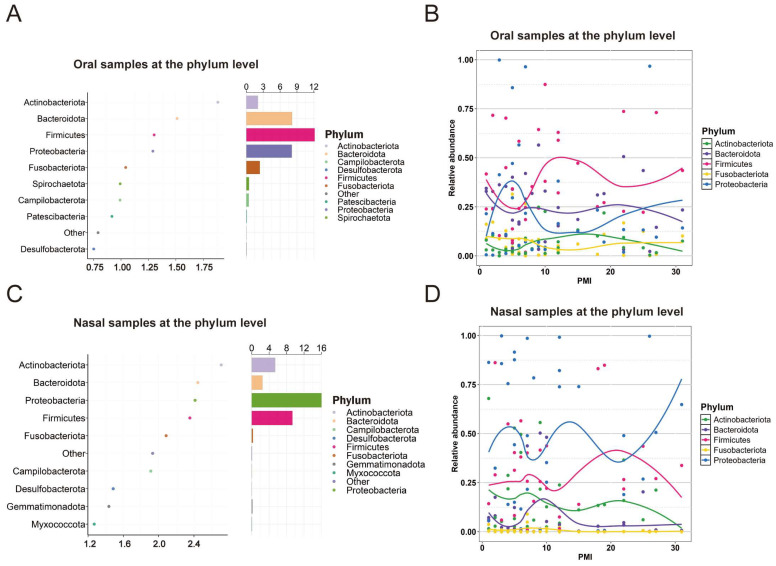
Phylum-level oral and nasal biomarkers for PMI estimation. (**A**) Top 10 phylum-level oral microbial biomarkers identified by the random forest model based on their abundance levels. (**B**) LOESS regression model of PMI based on the relative abundances of five selected oral microbial biomarkers. (**C**) Top 10 phylum-level nasal microbial biomarkers identified by the random forest model based on their abundance levels. (**D**) LOESS regression model of PMI based on the relative abundances of five selected nasal microbial biomarkers.

At the phylum level, both the oral and nasal groups shared the same biomarkers: *Firmicutes*, *Proteobacteria*, *Bacteroidota*, *Actinobacteriota*, and *Fusobacteriota* (Figure 3A,C). In the oral group, key turning points were observed on days 5, 12, and 22. From day 0 to day 5, all biomarkers, except *Proteobacteria*, showed a decreasing trend, while *Proteobacteria* exhibited an increasing trend. From day 6 to day 12, *Firmicutes* and *Actinobacteriota* increased, while *Proteobacteria* decreased. From day 12 to day 22, the trends reversed, with all phyla showing changes opposite to their earlier patterns. After day 22, *Firmicutes* and *Proteobacteria* increased, whereas *Bacteroidota* and *Actinobacteriota* declined (Figure 3B). In the nasal group, similar turning points and trends were observed, but the trends for *Proteobacteria* and *Firmicutes* were reversed compared to the oral group. Moreover, these two phyla exhibited opposite trends during different PMI stages within the nasal group (Figure 3D).

**Figure 4:**
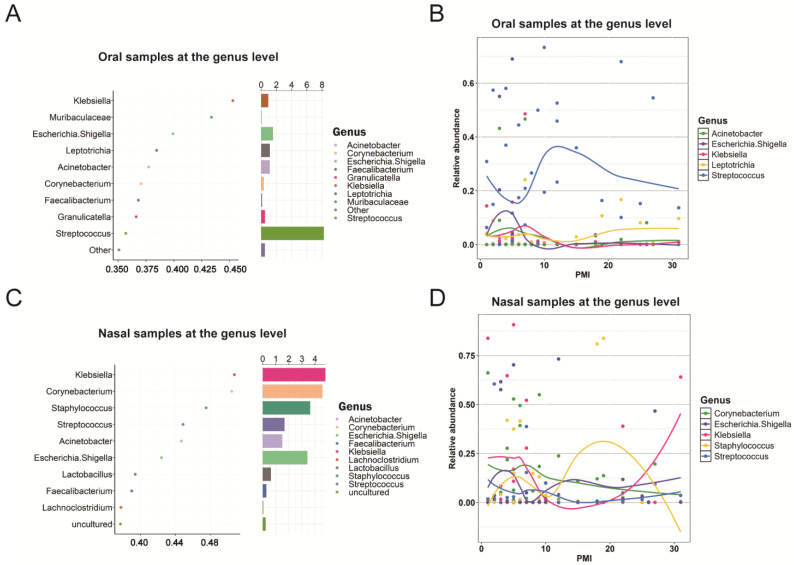
Genus-level oral and nasal biomarkers for PMI estimation. (**A**) Top 10 genus-level oral microbial biomarkers identified by the random forest model based on their abundance levels. (**B**) LOESS regression model of PMI based on the relative abundances of five selected oral microbial biomarkers. (**C**) Top 10 phylum-level nasal microbial biomarkers identified by the random forest model based on their abundance levels. (**D**) LOESS regression model of PMI based on the relative abundances of five selected nasal microbial biomarkers.

At the genus level, five biomarkers were identified in the oral group, including *Streptococcus*, *Escherichia-Shigella*, *Acinetobacter*, *Klebsiella*, and *Leptotrichia* (Figure 4A). The trend of *Streptococcus* mirrored that of *Firmicutes* at the phylum level, while the other genera showed an increasing trend from day 1 to day 7, followed by a decline (Figure 4B). For the nasal group, *Klebsiella*, *Corynebacterium*, *Staphylococcus*, *Escherichia-Shigella*, and *Streptococcus* were identified as biomarkers (Figure 4C). The trend of *Staphylococcus* mirrored that of *Firmicutes* at the phylum level, and *Escherichia-Shigella* displayed a trend similar to *Proteobacteria* at the phylum level (Figure 4D). Of note, while the oral and nasal groups showed similar turning points and trends, the trends and relative abundance of certain genera, such as *Proteobacteria* and *Firmicutes*, were reversed between the two groups.

Using the identified biomarkers, PMI inference models were constructed and evaluated based on the mean absolute error (MAE) and R^2^ values (Figure S1A–D). Among the four models, the oral genus-level and phylum-level models achieved the lowest MAE values for the training (MAE = 2.16 days) and testing datasets (MAE = 5.14 days). When biomarkers from both the oral and nasal groups were combined, the phylum-level model achieved an MAE of 2.66 days, while the genus-level model achieved an MAE of 2.26 days (Figure S1E,F).

### 2.5. Comparison of Samples from Frozen and Unfrozen Cadavers

In this study, 23 cadavers were stored at −20 °C within 12 h postmortem until dissection and sampling. To evaluate the potential impact of freezing on the microbiota, we compared the microbial communities of samples from frozen (Oral cavity-F and Nasal cavity-F) and unfrozen cadavers (Oral cavity-N and Nasal cavity-N).

**Figure 5:**
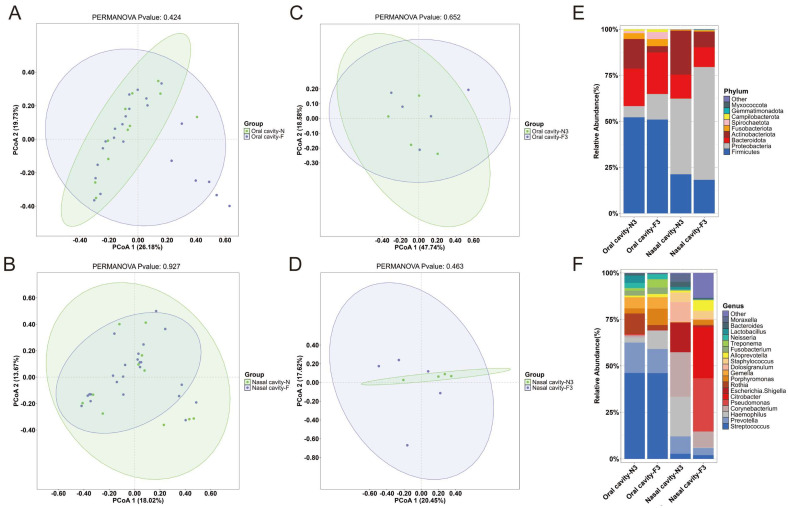
Impact of freezing on microbial communities of cadavers. (**A**) PCoA plot for oral microbiota in frozen versus unfrozen cadavers. (**B**) PCoA plot for nasal microbiota in frozen versus unfrozen cadavers. (**C**) PCoA plot for oral microbiota in frozen versus unfrozen cadavers in group D3. (**D**) PCoA plot for nasal microbiota in frozen versus unfrozen cadavers in group D3. (**E**,**F**) Phylum- and genus-level composition and relative abundance of dominant microbial members in frozen versus unfrozen cadavers in group D3.

The PCoA plots showed no significant clustering differences between the frozen and unfrozen samples (Figure 5A,B). Since Group D3 had a relatively balanced and comparable number of frozen and unfrozen samples, it was selected for further analysis. Consistent with the overall findings, the PCoA results for this group also demonstrated no significant differences (Figure 5C,D). At the phylum level, the frozen samples exhibited a higher relative abundance of *Proteobacteria* and a lower relative abundance of *Actinobacteriota* compared to the unfrozen samples, regardless of whether they were from the oral or nasal cavity (Figure 5E). At the genus level, the composition of the dominant genera in the nasal group showed substantial differences between the frozen and unfrozen samples (Figure 5F).

Using the method described in Section 2.4, we analyzed PMI-associated biomarkers in both frozen and unfrozen groups. At the phylum level in the oral group, both the frozen and unfrozen samples shared the same biomarkers: *Firmicutes*, *Proteobacteria*, *Bacteroidota*, *Actinobacteriota*, and *Fusobacteriota* (Figure S2A,C). The LOESS curves analysis indicated consistent trends between the two groups, although the relative abundance of *Proteobacteria* was lower in the unfrozen samples (Figure S2B,D). At the genus level, however, the biomarkers differed significantly between the two groups (Figure S3). In the nasal group, the frozen and unfrozen samples also shared common biomarkers at the phylum level: *Firmicutes*, *Proteobacteria*, *Bacteroidota*, and *Actinobacteriota* (Figure S4A,C). While the overall trends were similar, the relative abundances of these phyla varied between the frozen and unfrozen samples (Figure S4B,D). At the genus level, *Klebsiella* was the only biomarker common to both groups (Figure S5).

To provide a clearer overview of our key findings, we summarized the main results in Table 2.

## 3. Discussion

This study offers valuable insights into the dynamics of the oral and nasal microbial communities in both living individuals and human cadavers, highlighting their potential utility in PMI estimation. Our findings demonstrated distinct differences in microbial richness, diversity, and composition across the groups, influenced by factors such as host health status, decomposition stages, and environmental conditions.

Alpha and beta diversity metrics were used to compare microbial communities in healthy volunteers and human cadavers with varying PMIs. The Chao1 index showed a significantly higher species richness in healthy individuals than in cadavers, and the Shannon index reflecting oral and nasal diversity was also higher in the living. This may be due to the stable internal environment, adequate nutrition, and favorable conditions in the oral and nasal cavity during life, which support microbial survival and reproduction. After death, the loss of physiological functions leads to environmental changes, explaining the higher alpha diversity in healthy individuals than in cadavers [27]. The oral samples showed a higher species richness than the nasal samples, likely due to the mouth’s diverse nutrients and complex microenvironments, while the nose’s primary function and strict immune defenses limit microbial diversity [28]. The beta diversity analysis revealed clear clustering by sample site (oral vs. nasal) [29] but no significant clustering based on PMI, possibly due to individual variations within the PMI groups.

The analysis of microbial community composition across the 10 groups (Oral cavity-D1, Oral cavity-D2, Oral cavity-D3, Oral cavity-D4, Nasal cavity-D1, Nasal cavity-D2, Nasal cavity-D3, Nasal cavity-D4, Oral cavity-H, and Nasal cavity-H) revealed distinct patterns reflecting the health status and post-mortem changes. At the phylum level, *Proteobacteria*, *Firmicutes*, and *Bacteroidota* dominated in the oral cadaver groups, consistent with the findings from previous studies on human cadavers [30], rats [17], and mice [22]. In nasal cadaver groups, *Proteobacteria*, *Firmicutes*, and *Actinobacteriota* were predominant, with a particularly high abundance of *Proteobacteria*, aligning with our earlier study [31]. The higher *Actinobacteriota* levels in nasal samples likely reflect the nasal cavity’s greater exposure to environmental microbes [32]. In the healthy volunteers, *Actinobacteriota* was significantly more abundant in both the oral and nasal samples compared to those from cadavers, likely due to their preference for aerobic conditions, while decomposition favors anaerobic bacteria. Additionally, the nasal samples from the healthy individuals had a lower relative abundance of *Proteobacteria* compared to the cadavers, suggesting the post-mortem proliferation of *Proteobacteria*, which plays a crucial role in nutrient recycling during decomposition [33]. At the genus level, *Streptococcus* was dominant in both the healthy and cadaver oral groups, serving as a foundational pioneer species in the oral cavity due to its adaptability. In the healthy individuals, aerobic *Haemophilus* was prevalent in the oral samples, while anaerobic *Prevotella* dominated in the oral cadaver groups, reflecting a shift driven by the anaerobic and protein-rich environment of decomposition [34]. The nasal cadaver groups displayed genus-level variations across different PMIs, reflecting temporal microbial succession during decomposition as opportunistic and environmentally adaptive microbes progressively replace commensal species [32].

The random forest models identified PMI-associated biomarkers in the oral and nasal microbiomes. At the phylum level, *Firmicutes*, *Proteobacteria*, *Bacteroidota*, *Actinobacteriota*, and *Fusobacteriota* emerged as key biomarkers, consistent with previous studies [21]. These biomarkers exhibited non-linear abundance patterns, with turning points on days 5, 12, and 22. In the oral microbiome, *Firmicutes* and *Bacteroidota* dominated from day 0 to day 5 but gradually declined, while *Proteobacteria* steadily increased. These patterns align with previous studies [21,35] and may be driven primarily by changes in the environment and nutrient availability. The progressive depletion of oxygen may suppress the metabolic activity of anaerobic and facultative anaerobic bacteria like *Firmicutes* and *Bacteroidota*, whereas the aerobic and microaerophilic bacteria within *Proteobacteria* adapted and proliferated.

From day 6 to day 12, *Firmicutes* increased, aligning with previous findings [21], while *Bacteroidota* decreased and *Actinobacteriota* rose, consistent with the observations reported in [24]. In this period, the accumulation of decomposition byproducts, such as short-chain fatty acids and amino acids, favors *Firmicutes* [11], while the depletion of complex polysaccharides reduces *Bacteroidota* [12]. *Actinobacteriota* thrive on secondary metabolites, while *Proteobacteria* are inhibited under progressively anaerobic conditions. Interspecies resource competition and metabolic cooperation likely further influence these trends [36].

After day 12, the oral microbiome of the cadavers displayed distinct changes, with a turning point on around day 22. *Firmicutes* initially declined but later rebounded, while *Bacteroidota* increased before decreasing. *Actinobacteriota* showed a continuous decline, whereas *Proteobacteria* steadily increased. These shifts reflect the evolving decomposition environment. Specifically, *Firmicutes* experienced an early decline due to resource depletion and increasingly anaerobic conditions but later rebounded as decay-adapted taxa, such as *Clostridium*, proliferated under the putrefactive conditions. *Bacteroidota* likely utilized short-term decomposition products for initial growth but their growth decreased as the resources became exhausted. The persistent decline of *Actinobacteriota* suggests their limited adaptability to complex decomposition environments. In contrast, *Proteobacteria* used their metabolic flexibility and adaptability to anaerobic conditions to thrive and occupy the available ecological niches by relying on late-stage decomposition products.

In the nasal groups, similar trends and turning points as those in the oral groups were observed. However, the abundance patterns of *Proteobacteria* and *Firmicutes* were reversed. This divergence likely resulted from the environmental differences between the two cavities. The nasal cavity, an open system with higher oxygen levels, favors aerobic bacteria like *Proteobacteria* while inhibiting anaerobes like *Firmicutes*. In contrast, the hypoxic oral cavity provides a suitable environment for anaerobic bacteria [29]. Additionally, the nasal cavity’s immune defenses may limit anaerobic growth, promoting aerobic taxa instead.

At the genus level, the key biomarkers identified in the oral group included *Streptococcus*, *Escherichia-Shigella*, *Acinetobacter*, *Klebsiella*, and *Leptotrichia*. The abundance of *Streptococcus* mirrored the trends observed for *Firmicutes* at the phylum level, indicating that *Streptococcus* is a major PMI-associated genus within the *Firmicutes* phylum. Other biomarkers showed an initial increase from day 1 to day 7, followed by a decline, likely driven by the depletion of short-term decomposition products that initially supported their growth. In the nasal microbiome, *Klebsiella*, *Corynebacterium*, *Staphylococcus*, *Escherichia-Shigella*, and *Streptococcus* were identified as biomarkers, with *Staphylococcus* mirroring the trend of *Firmicutes* and *Escherichia-Shigella* and aligning with changes in *Proteobacteria*. This suggested that these genera are key representatives of their respective phyla in the nasal microbiome. While similar trends and turning points were observed in both the oral and nasal groups, the abundance of genera from *Proteobacteria* and *Firmicutes* showed opposite trends between the two environments, reflecting the oxygen-rich, open environment of the nasal cavity, which favors aerobic and facultative anaerobic taxa like *Proteobacteria*, compared to the hypoxic, closed oral cavity that supports anaerobic taxa like *Firmicutes* [28].

Using the identified biomarkers, PMI inference models were constructed. The models based on oral biomarkers at both the genus and phylum levels demonstrated superior accuracy, achieving the lowest MAE values in the training (MAE = 2.16 days) and testing datasets (MAE = 5.14 days). These findings indicate that the oral microbiota provide more reliable biomarkers for PMI estimation compared to the nasal microbiota. Furthermore, the MAE achieved by our model is comparable to that from a previous study utilizing a k-nearest neighbor (KNN) regression model based on phylum-level nasal and ear canal microbiome data, which estimated PMI with an MAE of approximately 55 accumulated degree days, equivalent to around three days [23]. It is worth noting that PMI inference models based on animal microbiomes are more common in the current research. For instance, Wang et al. analyzed oral microbiota from 21 rats sampled at seven time points within 168 h postmortem. Using genus-level microbial abundance and a random forest model, they achieved an R^2^ of 98.76% and an MAE of 6.93 ± 1.19 h [16]. In contrast, the lower accuracy of our model could be attributed to the human samples’ higher inter-individual variability and the study’s longer time span, with fewer samples collected at each time point.

Our study also examined the impact of freezing on postmortem microbiota dynamics by comparing the oral and nasal microbiota from frozen and non-frozen cadavers across varying PMIs. The results showed that freezing had minimal impact on the overall microbiota composition at the phylum level, consistent with a previous study [26]. However, freezing significantly altered the relative abundance of certain phyla, such as *Proteobacteria* and *Actinobacteriota*. This shift likely resulted from selective pressures induced by freezing, including low temperature and limited nutrient diffusion, which favor cold-tolerant taxa like *Proteobacteria* while suppressing taxa like *Actinobacteriota* that thrive in stable aerobic conditions. At the genus level, the freezing effects were more pronounced in the nasal microbiota than in the oral microbiota, indicating that the oral microbiota were less affected by temperature fluctuations. This suggests that oral microbiota may offer a more stable foundation for PMI estimation. Additionally, the PMI-associated biomarkers varied between the frozen and non-frozen groups, highlighting the necessity of accounting for temperature effects when selecting biomarkers for accurate PMI estimation.

We investigated the dynamics of the oral and nasal microbiota in both healthy individuals and human cadavers, identifying PMI-associated biomarkers and their nonlinear temporal trends. In addition, we developed random forest-based models for PMI prediction and examined the impact of freezing on cadaver microbiota. However, there were certain limitations to this study. First, the distribution of the frozen and non-frozen cadavers across the different PMIs was uneven, potentially introducing bias. Second, despite constructing PMI prediction models, the relatively high MAEs suggest limited model accuracy, likely owing to the uneven sample size across the PMIs and individual variability. Moreover, we did not explore the potential impact of environmental temperature on microbiota-based PMI estimation. Future research should aim to increase the sample size at each PMI and control environmental variables to improve the PMI inference models’ accuracy and precision.

## 4. Materials and Methods

### 4.1. Sample Collection

Sample collection was conducted in a controlled environment using sterilized equipment. The technicians adhered to stringent aseptic practices, including the use of sterile gloves, masks, and, protective clothing, to minimize contamination from the environment or personnel sources.

Samples of oral and nasal microbiota were collected from 10 healthy volunteers and 34 cadavers using sterile cotton swabs (Finegene Biotech, Shanghai, China). Healthy volunteers were recruited based on strict exclusion criteria, ensuring that individuals with dental pathologies (e.g., periodontitis, caries) or smoking habits—both of which could influence the oral microbiota—were not included. For the frozen cadavers, the time between death and refrigeration did not exceed 12 h. Separate swabs were used for each anatomical site, which were gently rotated and rubbed over the sampling area for 3–5 s to ensure adequate microbiota collection. The collected samples were immediately stored at −80 °C until further processing.

Written informed consent was obtained from all volunteers. The cadavers were acquired for scientific research through the documented consent of the donors and/or their legal next of kin. Ethical approval for the study was granted by the Ethics Committee of Fudan University (No. 2023C011).

### 4.2. DNA Extraction, Amplification and Sequencing

Microbial DNA was extracted from the collected swabs using the DNeasy PowerSoil kit (Qiagen, Hildesheim, Germany). The DNA concentration and integrity were measured using a NanoDrop 2000 spectrophotometer (Thermo Fisher Scientific, Waltham, MA, USA) and agarose gel electrophoresis. The V3-V4 hypervariable region of the microbial 16S rRNA gene was PCR-amplified in a 25 μL reaction using universal primer pairs (343F: 5′-TACGGRAGGCAGCAG-3′; 798R: 5′-AGGGTATCTAATCCT-3′). The reverse primer contained a sample barcode and both primers were connected with an Illumina sequencing adapter. Sequencing was conducted on an Illumina NovaSeq6000 with paired-end reads of 250 bases each (Illumina, San Diego, CA, USA; OE Biotech Company, Shanghai, China).

### 4.3. Data Analysis

The raw sequencing data, in FASTQ format, underwent quality trimming and filtering. Low-quality reads were removed, and DADA2 [37] was used to denoise, merge reads, and detect the chimeric sequences. These steps were performed using the default parameters of QIIME2 (https://qiime2.org/ (accessed on 11 September 2024)) [38]. Representative sequences for each ASV were selected through QIIME2, and annotations were carried out using the Silva database, version 138 (or Unite) (16s/18s/ITS rDNA), and the q2-feature-classifier with the default parameters. The microbial diversity within the groups was evaluated using alpha diversity metrics, including the Chao1 [39] and Shannon indices [40], which was calculated using QIIME2. Pairwise comparisons between the groups were conducted using the Wilcoxon test. Beta diversity, which indicates inter-group microbial composition variation, was analyzed using PCoA based on Bray–Curtis distance matrices. Group differences were statistically tested using the Adonis test, with the significant threshold set at *p* < 0.05.

### 4.4. Random Forest and LOESS Regression Modeling

For each anatomical site, the samples were randomly split into training (70%) and test datasets (30%). The relative abundances of bacterial taxa at the phylum or genus level were regressed against the cadavers’ PMIs using the “randomForest” package (https://cran.r-project.org/web/packages/randomForest/index.html (accessed on 12 December 2024)) in R [41]. Feature importance was assessed by ranking bacterial taxa based on their mean decrease in Gini values. Based on these rankings and the relative abundance data, potentially valuable PMI-associated biomarkers were identified. The performance of the PMI estimation models was evaluated by calculating the mean absolute error (MAE) and the goodness-of-fit (R²) values. Regression curves illustrating the relationship between biomarker changes and PMI were generated using the Locally Estimated Scatterplot Smoothing (LOESS) method. All statistical analyses and visualizations were conducted using R software (v4.4.1).

## 5. Conclusions

This study investigated the dynamics of the oral and nasal microbiota in healthy individuals and human cadavers, uncovering time-dependent changes in the microbial composition and abundance during decomposition. Potential PMI-associated biomarkers were identified, which demonstrating nonlinear changes with key turning points on days 5, 12, and 22. The random forest-based PMI prediction models showed promising results, laying the foundation for estimating PMI using microbiome data from these two body sites. A comparison between frozen and unfrozen cadavers unveiled the minimal impact of freezing on the microbial composition at the phylum level, although certain phyla, such as *Proteobacteria* and *Actinobacteriota*, showed altered relative abundances. Notably, the oral microbiota exhibited greater stability when subjected to temperature changes and superior predictive performance compared to the nasal microbiota. This work offers valuable insights into leveraging oral and nasal microbiome data for PMI estimation and highlights future directions to enhance model accuracy and understand microbial changes under varying environmental conditions.

## Figures and Tables

**Table 1:** Description of the cadavers and healthy volunteers analyzed in this study.

Group	Individual	PMI (Days)	Age (Years)	Gender	Cause of Death	Frozen	Season of Death
D1	S06	1	41	M	Craniocerebral injury	No	Spring
D1	S08	1	47	M	Coronary heart disease	No	Spring
D1	S09	2	37	F	Craniocerebral injury	No	Spring
D1	S10	2	48	M	Coronary heart disease	No	Spring
D1	S12	3	47	M	Drowning	No	Spring
D1	S40	3	60	F	Drug intoxication	Yes	Summer
D2	S17	4	49	M	Coronary heart disease	No	Spring
D2	S31	4	19	F	Traffic injury	Yes	Summer
D2	S05	5	80	M	Stomach tumor and lung infection	No	Winter
D2	S16	5	30	F	Craniocerebral injury	Yes	Spring
D2	S30	5	51	M	Traffic injury	Yes	Summer
D2	S24	5	65	M	Coronary heart disease	Yes	Spring
D2	S22	6	27	M	Sudden death	Yes	Spring
D2	S33	6	42	M	Electric shock	Yes	Summer
D2	S25	7	62	F	Septic shock and multiple organ failure	Yes	Spring
D2	S28	7	57	M	Sudden death	Yes	Spring
D2	S35	7	78	M	Aortoclasia	Yes	Summer
D3	S20	8	54	M	Hanging	Yes	Spring
D3	S21	9	80	M	Traffic injury	No	Spring
D3	S01	9	54	M	Craniocerebral injury	Yes	Winter
D3	S19	10	55	M	Acute pulmonary embolism	No	Spring
D3	S23	10	71	M	Traffic injury	Yes	Spring
D3	S18	12	51	M	Traumatic shock	No	Spring
D3	S14	12	23	F	Injury by fall from height	Yes	Spring
D3	S38	12	51	M	Unknown	Yes	Summer
D3	S02	15	64	M	Sudden cardiac death	Yes	Winter
D4	S27	18	46	M	Traffic injury	Yes	Spring
D4	S32	19	45	M	Traffic injury	Yes	Summer
D4	S04	22	87	F	Coronary heart disease	Yes	Winter
D4	S07	22	65	F	Coronary heart disease	Yes	Spring
D4	S11	25	28	M	Craniocerebral injury	Yes	Spring
D4	S34	26	74	M	Traffic injury	Yes	Autumn
D4	S39	27	42	M	Trauma	Yes	Summer
D4	S13	31	57	M	Coronary heart disease	No	Spring
H	1	-	20	M	-	-	-
H	2	-	24	F	-	-	-
H	3	-	28	M	-	-	-
H	4	-	27	M	-	-	-
H	5	-	26	F	-	-	-
H	6	-	34	F	-	-	-
H	7	-	28	M	-	-	-
H	8	-	31	M	-	-	-
H	9	-	23	F	-	-	-
H	10	-	22	F	-	-	-

**Table 2:** Summary of main results.

Group	Oral Cavity-H	Oral Cavity-D	Nasal Cavity-H	Nasal Cavity-D
Alpha diversity	Higher in healthy individual group than cadaver group; higher in oral group than nasal group			
Beta diversity	Oral vs. nasal: significant clustering Healthy individual vs. cadaver: no significant clustering Frozen vs. unfrozen: no significant clustering			
Dominant phyla	*Proteobacteria*, *Firmicutes*, and *Actinobacteriota*	*Proteobacteria*, *Firmicutes*, and *Bacteroidota*	*Proteobacteria*, *Firmicutes*, and *Actinobacteriota*	*Proteobacteria*, *Firmicutes*, and *Actinobacteriota*
	Frozen vs. unfrozen: frozen samples exhibited a higher relative abundance of *Proteobacteria* and a lower relative abundance of *Actinobacteriota* compared to unfrozen samples			
Dominant genera	*Streptococcus* and *Haemophilus*	*Streptococcus* and *Prevotella*	*Corynebacterium* and *Staphylococcus*	Variation across different PMI stages
	Frozen vs. unfrozen: the dominant genera in the nasal group showed substantial differences between frozen and unfrozen samples			
PMI-related biomarkers (phylum level)	-	*Firmicutes*, *Proteobacteria*, *Bacteroidota*, *Actinobacteriota*, and *Fusobacteriota* Key turning points were observed on days 5, 12, and 22	-	*Firmicutes*, *Proteobacteria*, *Bacteroidota*, *Actinobacteriota*, and *Fusobacteriota* Similar turning points and the trends for *Proteobacteria* and *Firmicutes* were reversed compared to the oral group
	*-*	Frozen vs. unfrozen: shared the same biomarkers: *Firmicutes, * *Proteobacteria, Bacteroidota, Actinobacteriota,* and *Fusobacteriota*	-	Frozen vs. unfrozen: shared the same biomarkers: *Firmicutes,* *Proteobacteria, Bacteroidota,* and *Actinobacteriota*
PMI-related biomarkers (genus level)	-	*Streptococcus*, *Escherichia-Shigella*, *Acinetobacter*, *Klebsiella*, and *Leptotrichia*	-	*Klebsiella*, *Corynebacterium*, *Staphylococcus*, *Escherichia-Shigella*, and *Streptococcus*
	*-*	Frozen vs. unfrozen: different biomarkers	-	Frozen vs. unfrozen: different biomarkers except for *Klebsiella*

## Data Availability

The data that support the findings of this study are available upon reasonable request from the corresponding author.

## Supplementary Materials

The following supporting information can be downloaded at: https://www.mdpi.com/article/10.3390/ijms26073432/s1.
