# Supplementary material for: Unveiling the Forensic Potential of Oral and Nasal Microbiota in Post-Mortem Interval Estimation
Source: Int J Mol Sci. 2025 Apr 6;26(7):3432. doi: 10.3390/ijms26073432 (PMC11989810; doi:10.3390/ijms26073432)
Supplement: Supplementary file 1 [file ijms-26-03432-s001.zip › ijms-3495514-supplementary.pdf]

Supplementary Materials

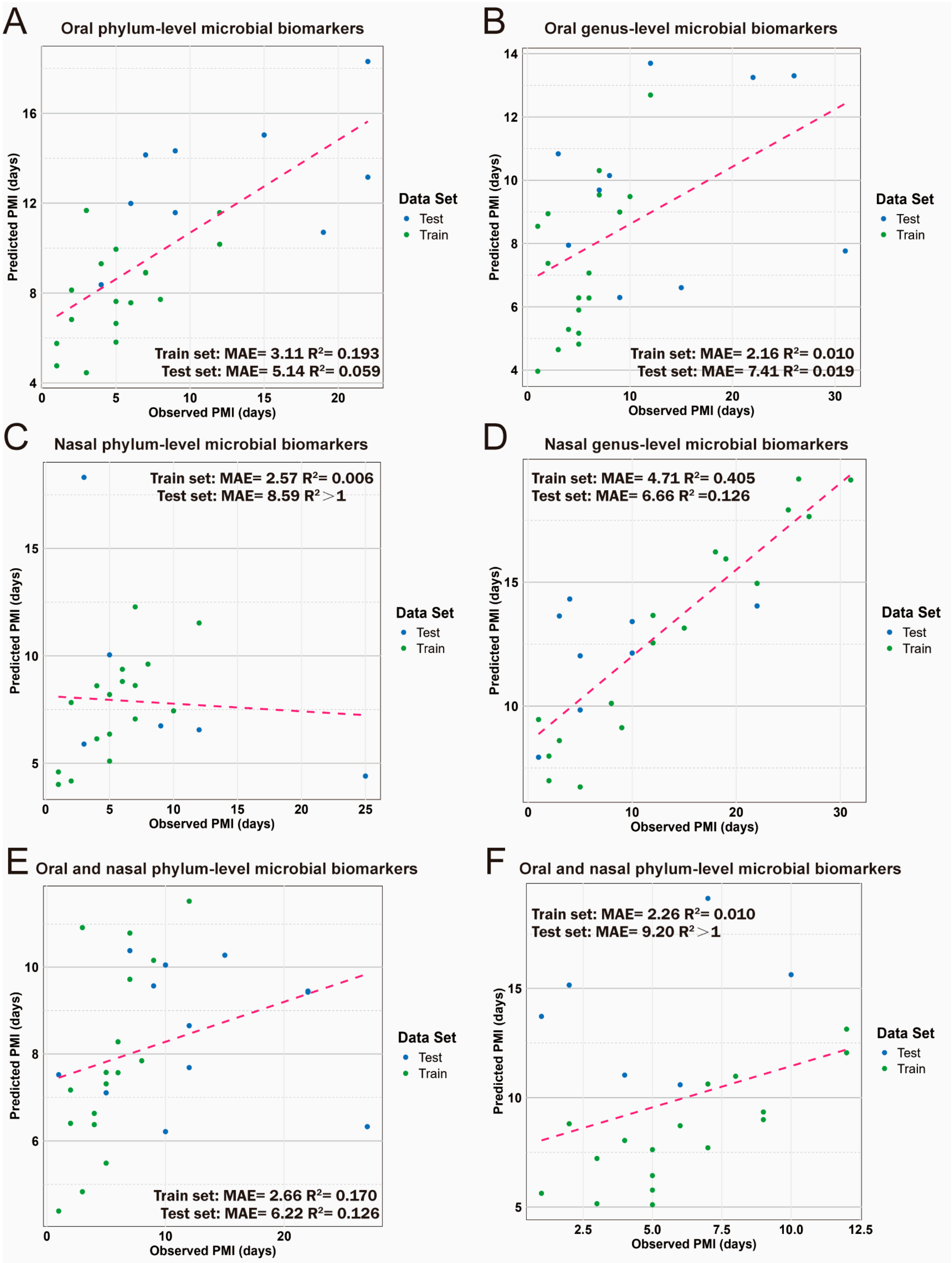

Figure S1. Post-mortem Interval (PMI) prediction models based on selected biomarkers. (**A**) Model constructed using oral phylum-level microbial biomarkers. (**B**) Model constructed using oral genus-level microbial biomarkers. (**C**) Model constructed using nasal phylum-level microbial biomarkers. (**D**) Model constructed using nasal genus-level microbial biomarkers. (**E**) Model combining oral and nasal phylum-level microbial biomarkers for PMI prediction. (**F**) Model combining oral and nasal genus-level microbial biomarkers for PMI prediction.

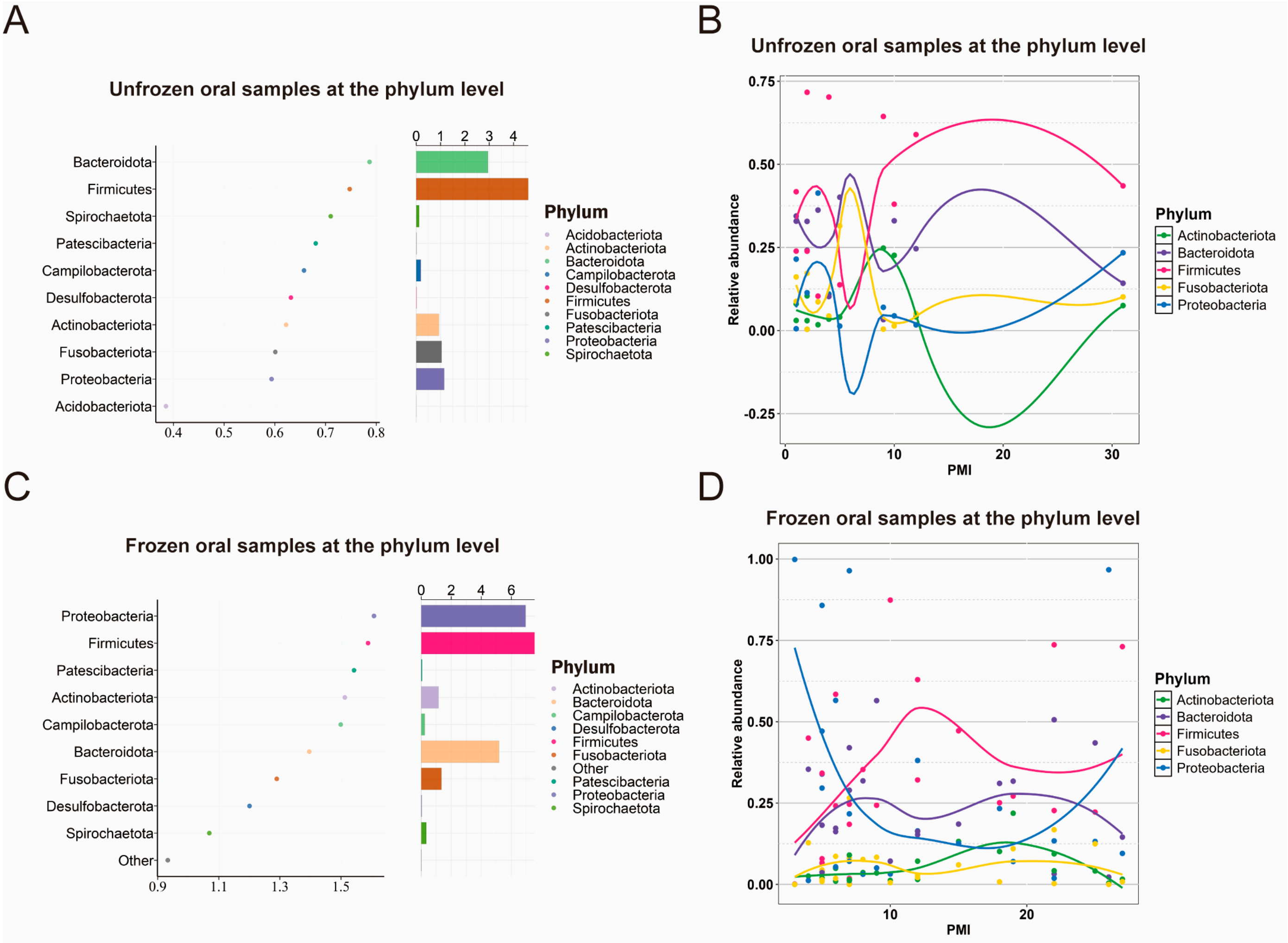

Figure S2. Phylum-level oral microbial biomarkers and LOESS regression curves in frozen versus unfrozen cadavers. (**A**) Top 10 phylum-level oral microbial biomarkers in unfrozen cadavers identified by the random forest model and their abundance levels. (**B**) LOESS regression model of PMI based on the relative abundances of selected five biomarkers. (**C**) Top 10 phylum-level oral microbial biomarkers in frozen cadavers identified by the random forest model and their abundance levels. (**D**) LOESS regression model of PMI based on the relative abundances of selected five biomarkers.

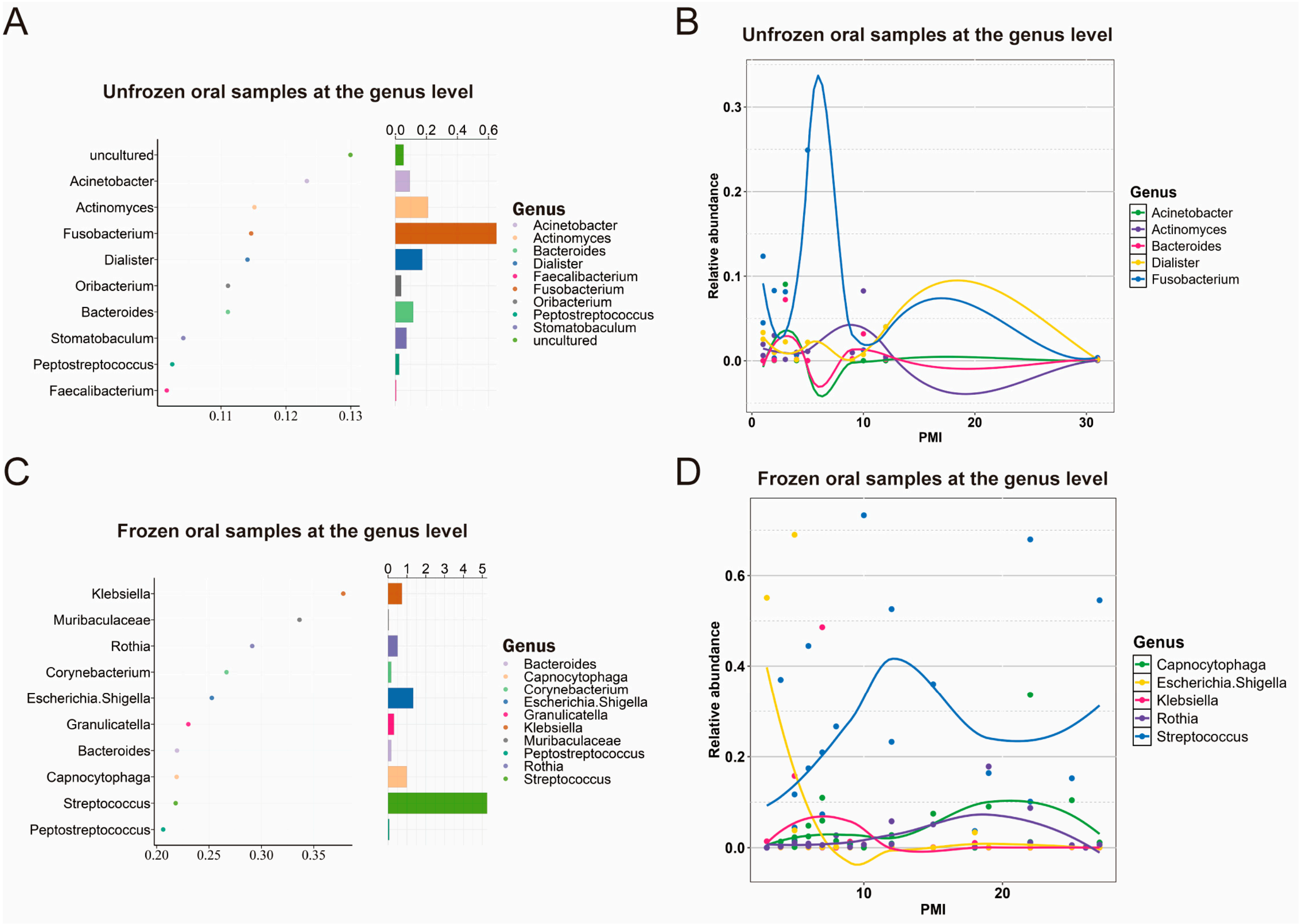

Figure S3. Genus-level oral microbial biomarkers and regression models in frozen versus unfrozen cadavers. (**A**) Top 10 genus-level oral microbial biomarkers in unfrozen cadavers identified by the random forest model and their abundance levels. (**B**) LOESS regression model of PMI based on the relative abundances of selected five biomarkers. (**C**) Top 10 genus-level oral microbial biomarkers in frozen cadavers identified by the random forest model and their abundance levels. (**D**) LOESS regression model of PMI based on the relative abundances of selected five biomarkers.

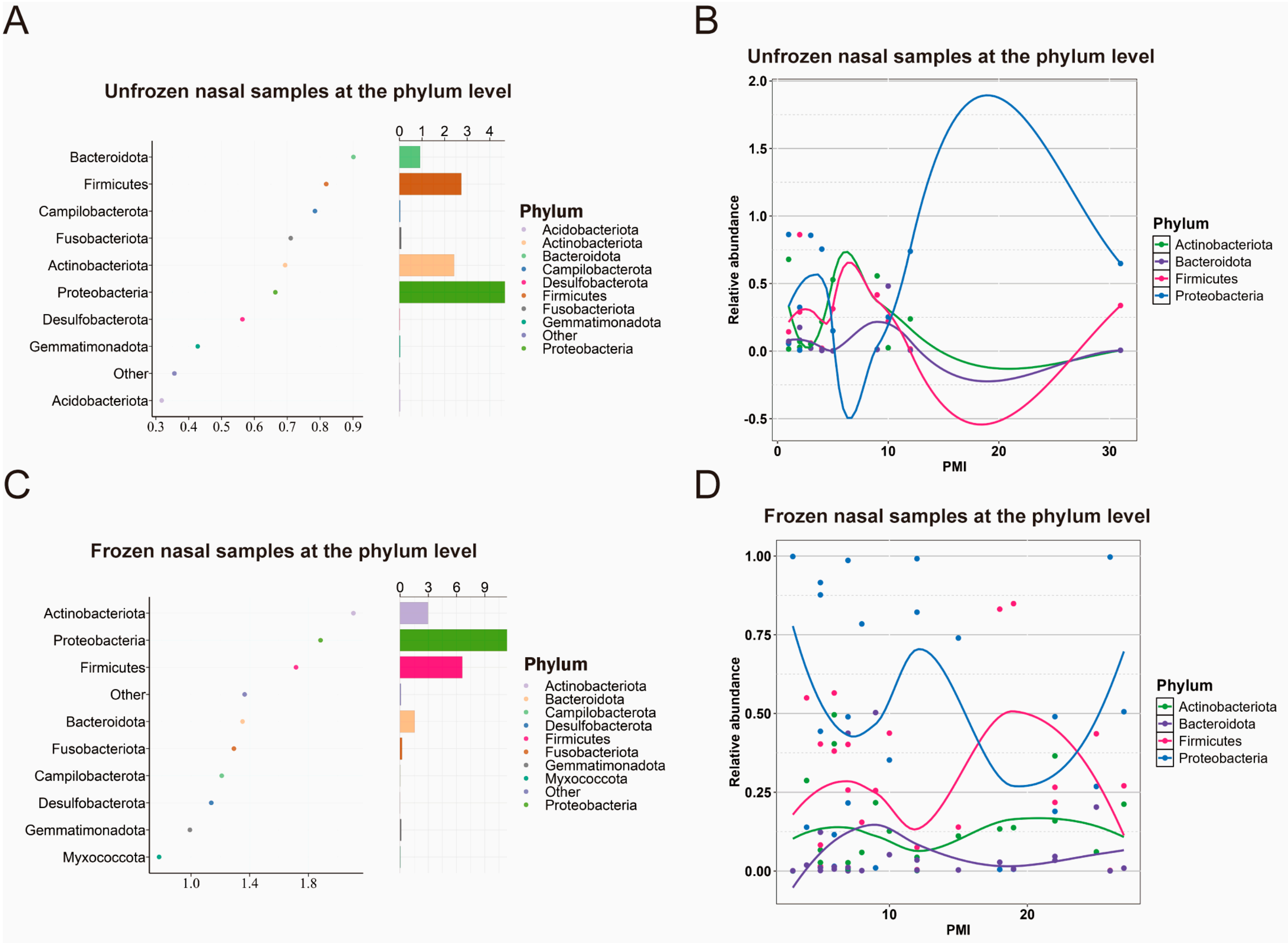

Figure S4. Phylum-level nasal microbial biomarkers and regression models in frozen versus unfrozen cadavers. (**A**) Top 10 phylum-level nasal microbial biomarkers in unfrozen cadavers identified by the random forest model and their abundance levels. (**B**) LOESS regression model of PMI based on the relative abundances of selected four biomarkers. (**C**) Top 10 phylum-level nasal microbial biomarkers in the frozen cadavers identified by the random forest model and their abundance levels. (**D**) LOESS regression model of PMI based on the relative abundances of selected four biomarkers.

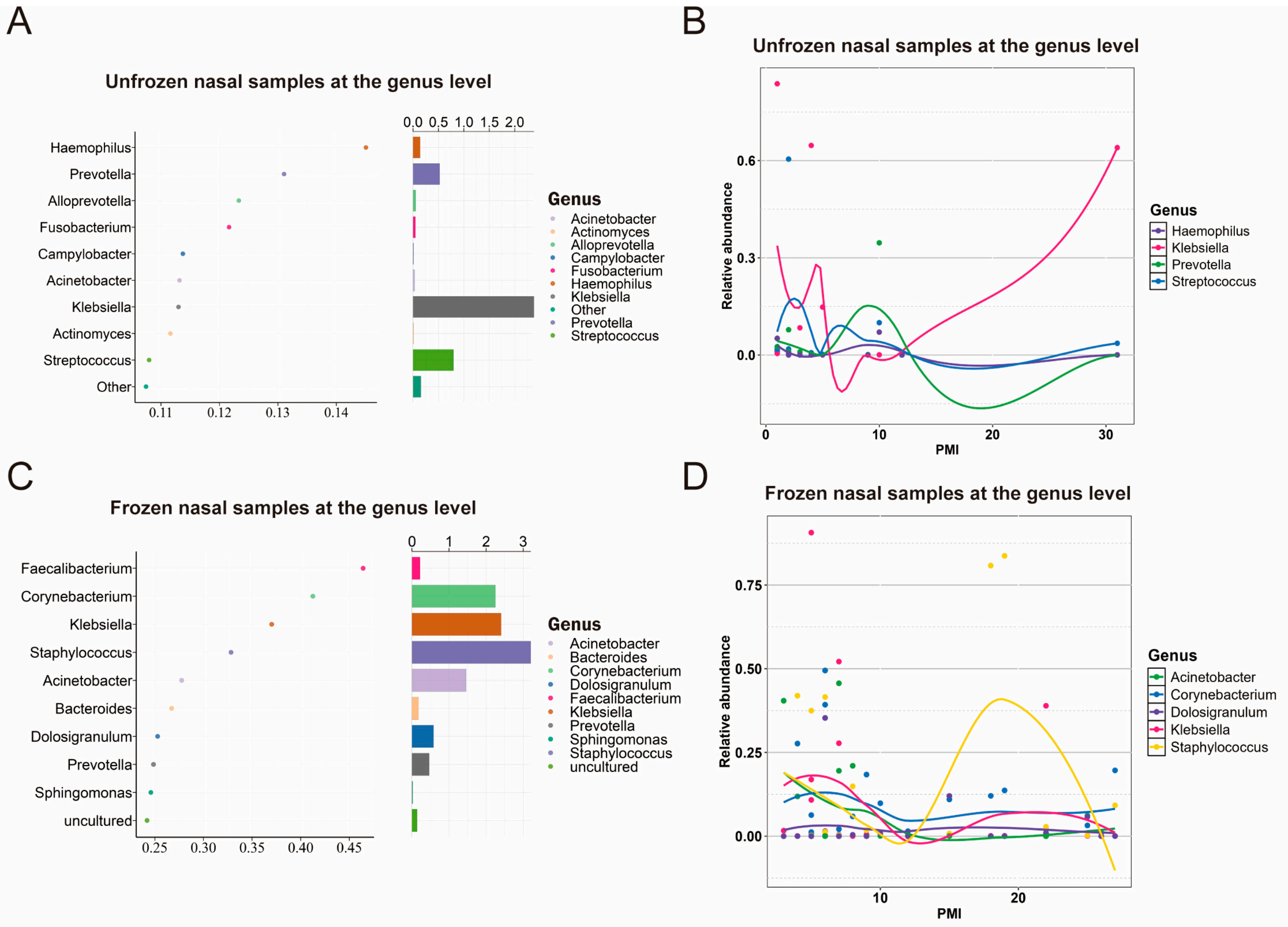

Figure S5. Genus-level nasal microbial biomarkers and regression models in frozen versus unfrozen cadavers. (**A**) Top 10 genus-level nasal microbial biomarkers in unfrozen cadavers identified by the random forest model and their abundance levels. (**B**) LOESS regression model of PMI based on the relative abundances of selected four biomarkers. (**C**) Top 10 genus-level nasal microbial biomarkers in the frozen cadavers identified by the random forest model and their abundance levels. (**D**) LOESS regression model of PMI based on the relative abundances of selected five biomarkers.
